# Arthroscopic foveal repair with suture anchors for traumatic tears of the triangular fibrocartilage complex

**DOI:** 10.1186/s12891-022-05588-z

**Published:** 2022-07-04

**Authors:** Kuang-Ting Yeh, Wen-Tien Wu, Jen-Hung Wang, Jui-Tien Shih

**Affiliations:** 1 Department of Orthopedics, Hualien Tzu Chi Hospital, Buddhist Tzu Chi Medical Foundation, Hualien, Taiwan; 2 School of Medicine, Tzu Chi University, Hualien, Taiwan; 3 Department of Medical Research, Hualien Tzu Chi Hospital, Buddhist Tzu Chi Medical Foundation, Hualien, Taiwan; 4 Department of Orthopaedic Surgery, Taoyuan Armed Forces General Hospital, NO. 168, Zhongxing Road, Longtan Dist, Taoyuan City, 32551 Taiwan

**Keywords:** Arthroscopic repair, Triangular fibrocartilage complex, Traumatic tears, Suture anchors

## Abstract

**Background:**

Foveal tears of the traumatic triangular fibrocartilage complex (TFCC) are the most commonly neglected high-energy injuries of the wrist joint, and the patients with such tears often experience unrecovered ulnar-sided wrist pain and poor wrist function. This study investigated the functional outcomes of patients who underwent arthroscopic repair of foveal TFCC tears with suture anchors and adjuvant platelet-rich plasma (PRP) injections after the surgery.

**Methods:**

From September 2014 to August 2018, 156 men and 45 women with diagnoses of foveal TFCC tears without wrist fractures underwent arthroscopic repair by using the outside-in method with 1.3-mm suture anchors and subsequent PRP injection. After surgery, splinting was applied for 6 weeks, and the patients underwent rehabilitation, re-examination, and follow-up at our clinic. The patients’ wrist functional scores and grip strength data were retrospectively collected.

**Results:**

The mean follow-up period was 32.6 months, and the mean age was 26.7 years. The mean modified Mayo wrist score improved from 48.5 ± 2.6 to 82.4 ± 2.5, whereas the mean Disabilities of the Arm, Shoulder and Hand (DASH) score decreased from 39.2 ± 6.7 to 10.6 ± 7.5. Overall, the wrist functions of 186 (92.5%) of the patients were satisfactory according to their modified Mayo wrist scores, and the patients with satisfactory scores returned to sports or work activities. These patients retained normal ranges of motion, and their average grip strength in the affected hand was restored to at least 85% of that of the other hand.

**Conclusions:**

According to the postoperative 25–36 months surgical results of our study, arthroscopic repair with adjuvant PRP injections is a satisfactory method of repairing early foveal tears of the TFCC and can enhance wrist function by relieving pain and increasing tolerance for work or sports.

## Introduction

The triangular fibrocartilage complex (TFCC) complex in the wrist consists of the central fibrocartilage, dorsal and palmar distal radioulnar ligaments, sheath of the extensor carpi ulnaris (ECU) tendon, ulnar collateral ligaments, and ulnocarpal ligaments. It functions as the center of forearm rotation and enables the carpus to smoothly rotate with the radius around the ulna [1–5]. The TFCC has three crucial biomechanical functions: transmission of force from the wrist to the forearm, stabilization of the distal radioulnar joint (DRUJ) when the distal radioulnar ligaments become taut, and stabilization of the ulnar carpus through the ulnocarpal ligament complex [6, 7]. Because the TFCC is vulnerable to axial loads and shear forces, traumatic tears often occur in accidents when a pronated, hyperextended wrist quickly collides with the ground, or a sudden strong distraction force causes dislocation of the ulna [8]. The most common symptoms of TFCC injury are prominent ulnar-sided wrist pain and grip weakness, especially during work or sport. When the fovea of the TFCC is disrupted, the resulting instability of the wrist region can cause progressive arthritic changes in the wrist or intercarpal joints as well as motion limitation, grip weakness, and significant loss of wrist function. Directly increasing blood supply to the TFCC is crucial to the success rate of the repairing or reconstructing methods. The central area is avascular, and injuries to this area is often treated with debridement; by contrast, the peripheral and foveal regions of the TFCC receive sufficient blood supply, and injuries to these areas are often treated with surgical repair with the expectation of favorable postoperative functional outcomes [8]. The repair of foveal TFCC tears under arthroscopy is often challenging but may be a crucial step in the successful restoration of DRUJ function [9]. In addition to surgical repair, regenerative materials may be applied to accelerate the healing of the injured tissue. Platelet-rich plasma (PRP) may alleviate pain, improve patients’ functional outcomes, and prevent postoperative retear in arthroscopic rotator cuff repair [10] and may also be beneficial to the surgical outcomes of patients with wrist fractures and trapeziometacarpal arthritis [11]. The purpose of this study was to investigate the postoperative 24 months functional outcomes, namely the grip strength, modified Mayo wrist scores, and Disabilities of the Arm, Shoulder and Hand (DASH) scores, of patients who underwent our method of foveal repair using suture anchors with adjuvant PRP injections.

## Material and methods

The experimental protocol was established in accordance with the ethical guidelines of the Declaration of Helsinki and was approved by the local human ethics committee, and our findings are herein reported in accordance with the Strengthening the Reporting of Observational Studies in Epidemiology (STROBE) guidelines. The inclusion criteria were as follows: 1. diagnosis of traumatic foveal TFCC tears and subsequent arthroscopic repair between September 2014 and August 2018 and 2. a follow-up period of at least 18 months. The exclusion criteria were as follows: 1. Concomitant major injuries to the upper limbs or lower extremities, 2. an accident-to-surgery interval of over 12 weeks, and 3. degenerative changes in the TFCC detected using X-rays and magnetic resonance imaging (MRI). We collected the following data for each included patient: the cause of trauma, the range of motion arc angle and grip strength of the injured wrist, the percent difference between the grip strength of the injured wrist and the uninjured wrist, the modified Mayo wrist score, the DASH score, and postoperative complications (such as surgical site infections or exacerbated ulnar-sided wrist pain) during the follow-up period.

**Fig. 1:**
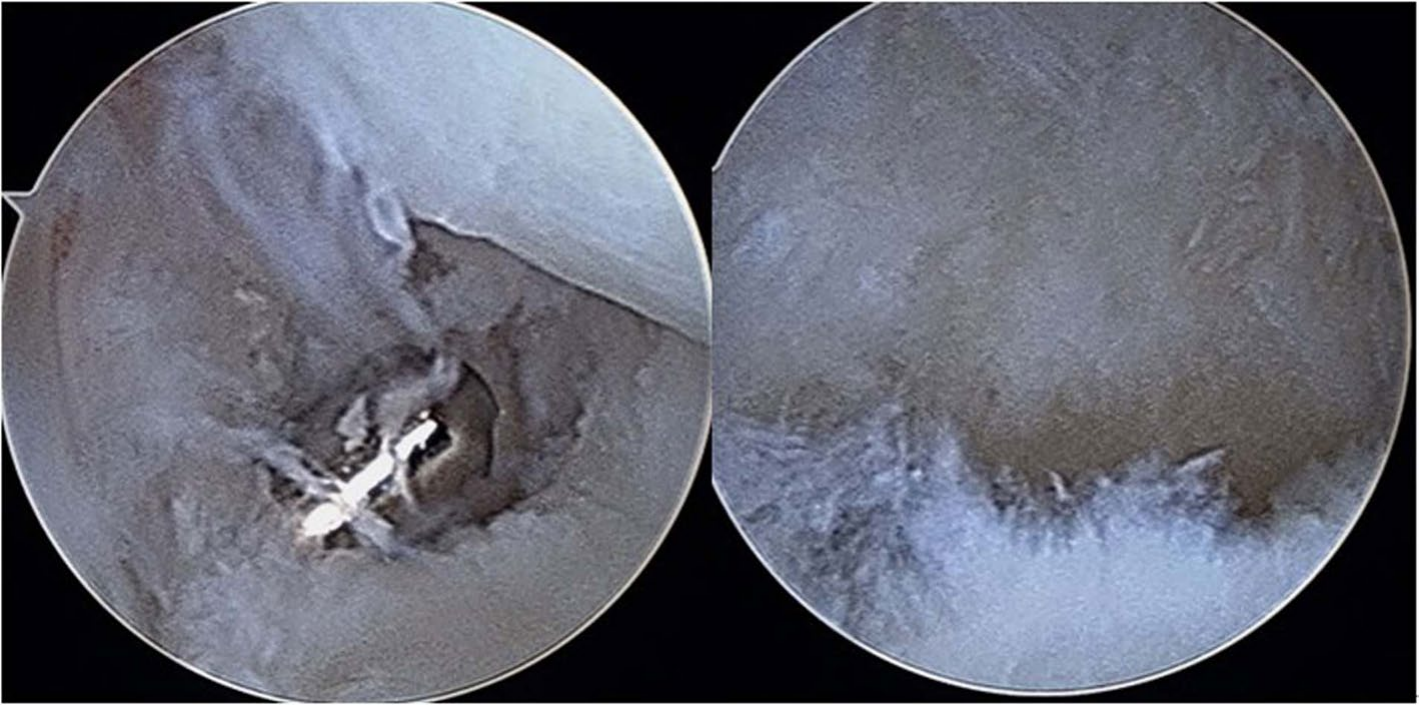
A Twenty-four-year male experienced fovea tear of TFCC due to falling down accident. It demonstrated tear post debirded with power shaver

We diagnosed foveal TFCC tears according to the patients’ physical examinations and symptoms, which included intolerable ulnar-sided wrist pain and decreased grip strength during work or sports activities, tenderness over the ballotable area of the ulna (between the triquetrum and the ulnar styloid), positive ulnar compression tests (Fig. 1), and positive piano key signs with full pronation and easily depressed ulnar protrusions. Each patient underwent a basic X-ray examination to identify any bone fractures or ulnar variance indicative of possible degenerative changes. All the patients who were suspected of having TFCC foveal tears were examined using MRI. All the patients included in this study had received at least 6 weeks of nonoperative treatment (protective splints) without experiencing alleviation of their symptoms. Arthroscopic repair of the TFCC was arranged for the patients who had prominent ulnar-sided wrist symptoms and failed to respond to conservative treatment for foveal TFCC tears detected through MRI. The patients’ TFCC tears were classified using the Atzei–European Wrist Arthroscopy Society (Atzei–EWAS) classification according to the findings of wrist arthroscopy [12].

### Surgical procedure

Wrist arthroscopy was performed under general anesthesia with an upper-arm tourniquet applied at a pressure of approximately 250 mmHg. After sterile preparation and draping, the patient’s hand was placed in a tower traction device with a force of approximately 10 to 15 lb. From a dorsal approach, the 3–4 and 6R portals were used for arthroscopy and power shaving. The 6R portal was created through longitudinal incision near the dorsal border of the ECU to help us more easily approach the prestyloid recess from the radiocarpal joint. We used the power shaver to release the TFCC from the capsule, thus enabling us to clearly approach even deep tears in the foveal region. Peripheral and deep tears of the TFCC were clearly visible through the 3–4 portal after the TFCC was released, and instability was assessed using a hook test with a probe. We further confirmed the presence of TFCC tears by squeezing the dorsal and volar sides of the DRUJ capsule and checking whether the DRUJ intra-articular fluid was forced into the radiocarpal space [13]. A power burr was then inserted through the 6R portal to debride and prepare the foveal region for the subsequent insertion of the suture anchor, and the operation field was visible through the 3–4 portal after adequate shaving was performed in the previous step.

**Fig. 2:**
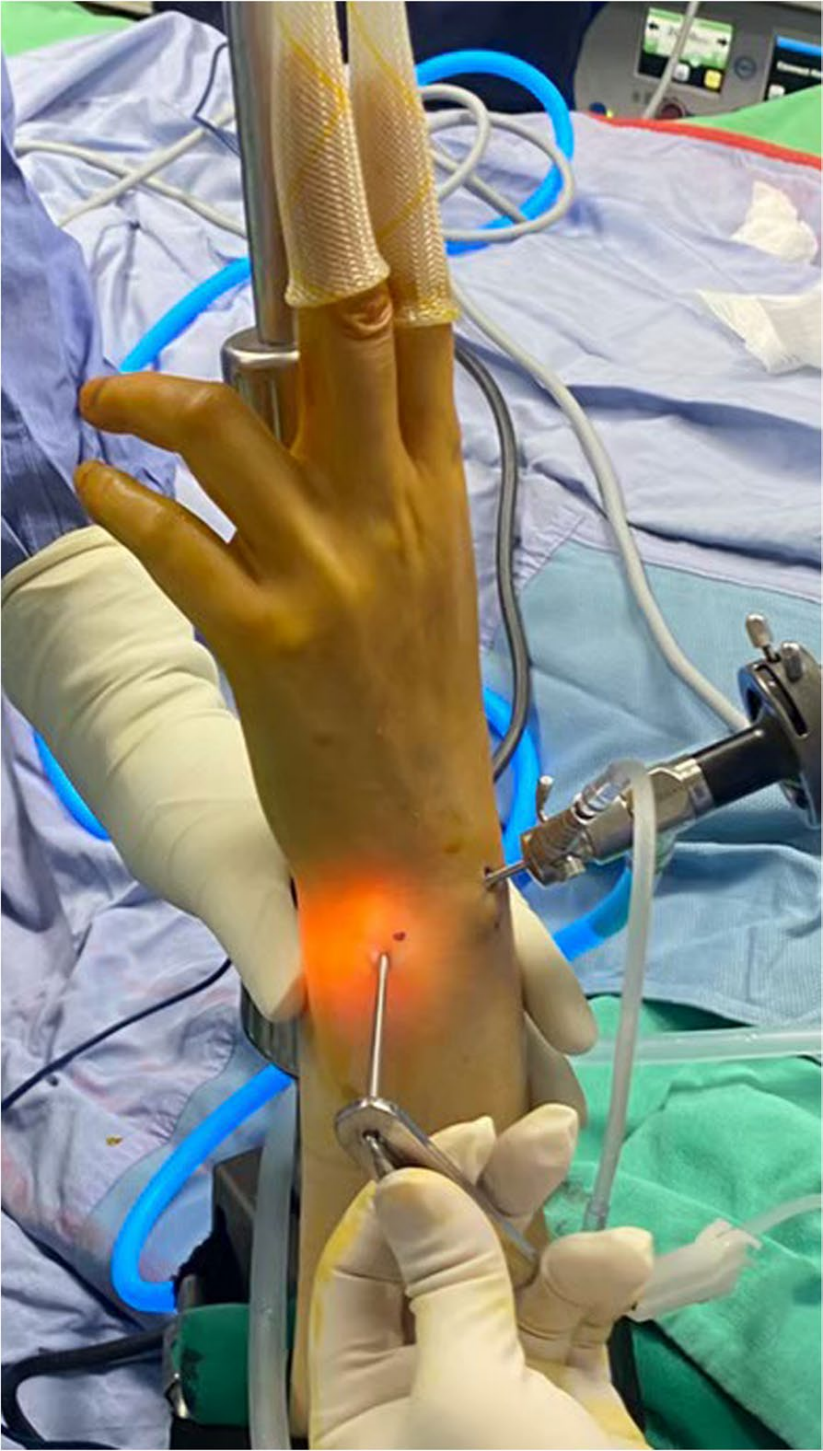
By way of ECU through the 6R portal, we applied the sheath of the 1.3 mm suture anchor to the fovea of the ulnar head

**Fig. 3:**
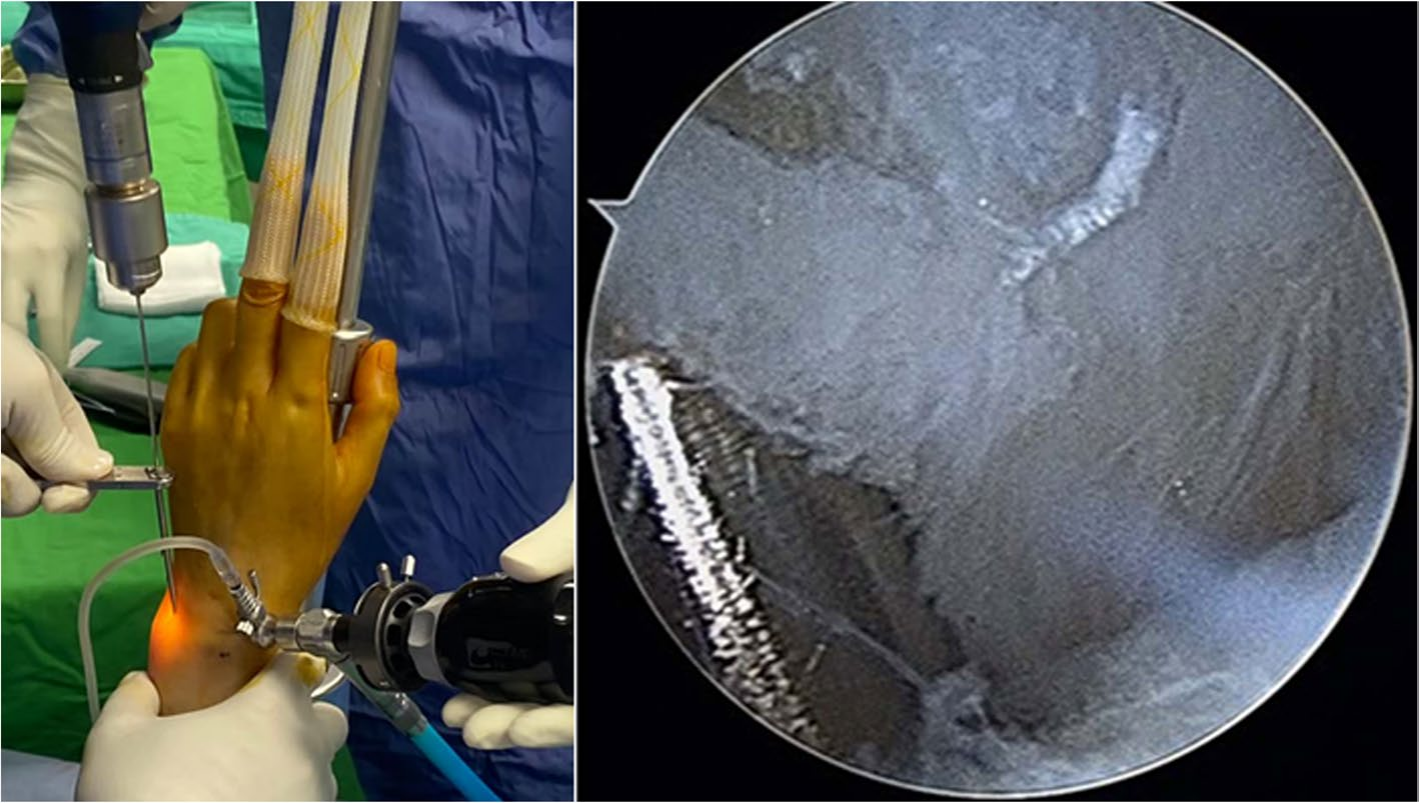
We applied a 1.4-mm K-wire to create a bony hole around the foveal region inside the sheath of the 1.3 mm suture anchor

**Fig. 4:**
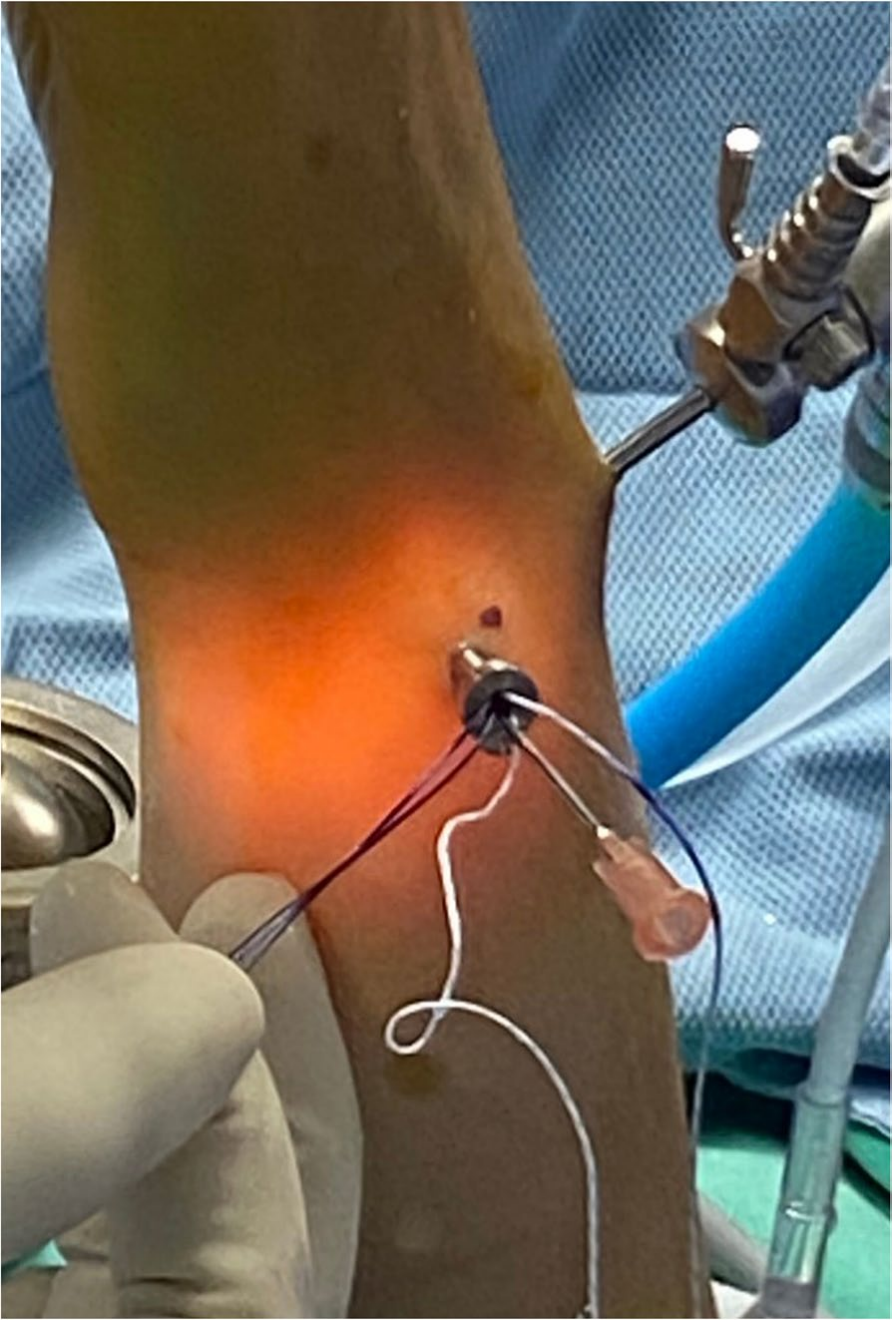
We inserted a 2.0 mm metal cannula through the 6R portal in prevention of surrounding soft tissue unnecessary interposition

**Fig. 5:**
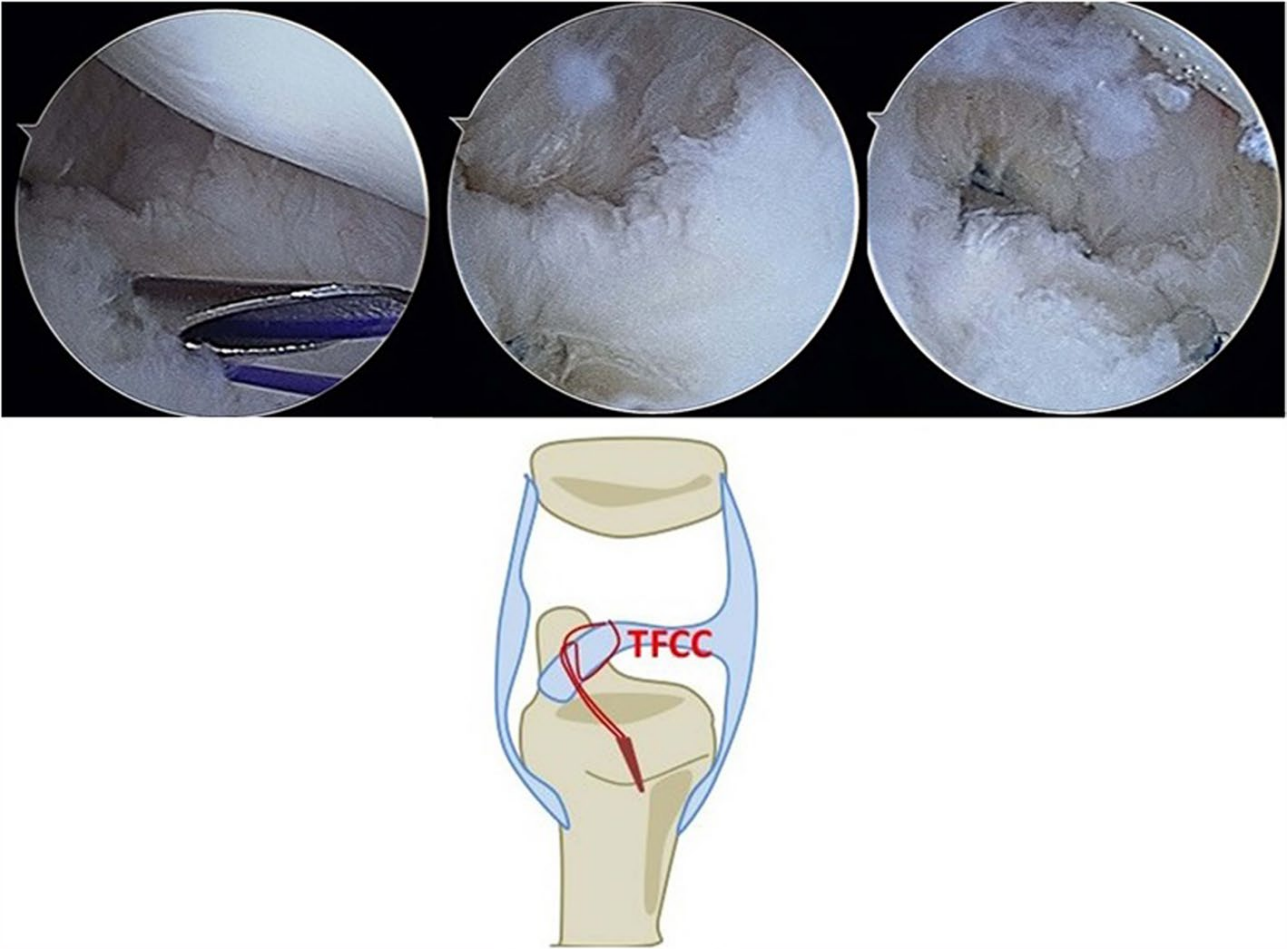
We applied the outside-in suture method to repair the tears with the suture anchor and confirmed that the TFCC was proximal reattached to fovea side well through the arthroscopic examination

The sheath (Fig. 2) of the 1.3-mm suture anchor (CONMED, Y-Knot Flex All-Suture Anchor, NY, USA) was first inserted through the 6R portal by the volar border of ECU tendon to enable us to locate the fovea of the ulnar head, and a 1.4-mm K-wire was used to create a bony hole around the foveal region inside the sheath (Fig. 3). The anchor was then applied through the sheath. We consistently evaluated the strength of the anchor to ensure stability. A metal cannula (2.0 mm; Fig. 4) was inserted through the 6R portal by using a suture anchor guide to ensure that the suture would be placed through the same portal without soft tissue interposition. The outside-in method was used to repair the tears with the suture anchor (Fig. 5). The wrist traction was then released, and the attachment between the repaired TFCC and the fovea was tightened using 3 knots tied using a knot pusher in the neutral forearm position with the ulnar head reduced to the sigmoid notch of the distal radius. The remaining suture tails were then secured to the ulnar capsule with 2 knots for peripheral augmentation. The knots were then placed inside the ECU tendon sheath to prevent skin irritation. The procedure combined foveal and peripheral TFCC repair. After the TFCC repair was completed, the PRP (Regen Lab SA, NY, USA) was prepared and injected into the wrist joint through the 6R portal. The surgeon then removed the arthroscope and all other arthroscopic instruments from the wrist and sutured the portal sites with 4–0 nylon sutures. Finally, a bulky sterile dressing was wrapped around the wrist, and the pneumatic tourniquet was deflated.

### Postoperative care, rehabilitation

A wrist brace was used to fix the wrist in a neutral position for 6 weeks. The subsequent occupational hand therapy program involved active and passive range of motion and isometric exercises. After 1 month of occupational therapy, the patients progressed to therapy that incorporated strengthening and work simulation exercises.

### Functional recovery evaluation

All the patients were retrospectively evaluated and followed up at the outpatient clinic by Dr. Shih. All the functional parameters were measured preoperatively and postoperatively (at the last follow-up) by Dr. Yeh. All the grip strength and range-of-motion measurements were obtained using a digital hand dynamometer (TTM Dynamometer, Tsutsumi, Tokyo, Japan) and a goniometer, respectively. The patients’ modified Mayo wrist scores were used to assess pain, active flexion/extension arc as a percentage of that of the opposite side, grip strength as a percentage of that of the opposite side, and the ability to return to regular employment or activities. Modified Mayo wrist scores range from 0 to 100 points, with scores of 90–100, 80–89, 65–79, and < 65 points corresponding to “excellent,” “good,” “fair,” and “poor” wrist function, respectively [14]. The DASH questionnaire, which consists of 21 items evaluating difficulty with specific tasks; 5 items evaluating symptoms; and 4 items evaluating social function, work function, sleep, and confidence, is scored from 0 to 100, with higher scores indicating poorer upper-extremity function [15, 16]. The patients’ postoperative complications were assessed using the International Consortium for Health Outcome Measurement Complications in Hand and Wrist conditions classification (ICHOW) standard set for classifying hand and wrist conditions [17].

### Statistical analysis

SPSS for Windows, version 23.0 (IBM, Armonk, NY, USA), was used for statistical analysis. When the effect size, level of significance, and power were set to 0.2, 0.05, and 0.80, respectively, the estimated sample size was at least 199, indicating that our sample size was sufficient. An a priori/post hoc power analysis was performed. An independent *t* test was used to compare the demographic data and preoperative conditions between the male and female patients. A paired *t* test was used to compare the patients’ preoperative and postoperative function scores. All the reported *P* values were two-sided, and a Bonferroni-corrected *P* value of < 0.01 was considered statistically significant. The continuous data were checked for normal distribution.

## Results

A total of 202 patients underwent arthroscopic repair from September 2014 to August 2018 at our hospital. One of them was lost to follow-up because they died in a car accident. We analyzed the data of the remaining patients, namely 156 men and 45 women with a mean age of 26.7 ± 4.2 years (Table 1). The causes of trauma were accidental falls for 117 of the patients, traffic accidents for 36, and military training accidents for the remaining 48. The mean interval from accident to repair was 8.2 weeks (range: 5–12 weeks). All the patients experienced intolerable ulnar-sided wrist pain and decreased grip strength during work or sports activities, and 83 of them experienced tenderness over the ballotable area of the ulna. Of the included patients, 62 had positive ulnar compression tests, and 51 exhibited positive piano key signs.

**Table 1:** Demographics (*N* = 201)

	Male	Female	Total	*P* value
*N*	156	45	201	
Age	25.8 ± 4.8	27.8 ± 5.4	26.7 ± 4.2	0.244
Mayo modified wrist scores (Preop)	51.0 ± 4.7	46.6 ± 5.8	48.5 ± 2.6	0.152
Disability of the Arm, Shoulder, and Hand scores (Preop)	36.3 ± 4.8	43.1 ± 5.9	39.2 ± 6.7	0.177
Grip strength ratio (%) (Preop)	41.1 ± 2.4	37.2 ± 3.2	38.6 ± 2.3	0.263

Data are presented as *N* values or means ± standard deviations

^*^A *P* value of < 0.05 was considered statistically significant

None of the patients had bony fractures, and the mean ulnar variance was 0.6 ± 0.4 mm. Overall, 78 and 123 of the patients had Atzei–EWAS classification class II and class III foveal tears, respectively, confirmed through wrist arthroscopy. None of the patients had interosseous ligament or carpal ligament tears.

The mean postoperative follow-up period was 32.6 months (range: 25–36 months). None of the patients complained of moderate to severe wrist pain during their daily activities. The mean preoperative flexion/extension and pronation/supination arcs were 135.0° (range: 125°–170°) and 126.0 (range: 105°–136°), respectively, and the corresponding measurements at the last follow-up were 132.0° (range: 113°–164°), and 122.0 (range: 100°–132°), respectively. The mean modified Mayo wrist score improved from 48.5 ± 2.6 preoperatively to 82.4 ± 2.5 postoperatively; the mean DASH score decreased from 39.2 ± 6.7 preoperatively to 10.6 ± 7.5 postoperatively (Table 2). Of the 201 patients’ wrists, 4 were rated “excellent,” 182 were “good,” and 15 were “fair” according to the modified Mayo wrist scores. The average grip strength ratio improved from 38.6% ± 2.3% preoperatively to 85.4% ± 2.8% postoperatively (Table 2). In total, 15 (7.5%) of the patients experienced ICHOM grade I complications (mild ulnar-sided wrist pain) during work or exercise. No postoperative infections nor other complications necessitating further surgical correction occurred during the follow-up period. Overall, 186 of the 201 patients (92.5%) exhibited satisfactory outcomes and returned to sports or work activities.

**Table 2:** Preoperative to postoperative changes in functional assessments (*N* = 201)

Item	Pre-OP	Post-OP	Diff	*P* value
Mayo modified wrist scores	48.5 ± 2.6	82.4 ± 2.5	32.7 ± 3.1	< 0.001*
Disability of the Arm, Shoulder, and Hand scores	39.2 ± 6.7	10.6 ± 7.5	28.4 ± 6.8	< 0.001*
Grip strength ratio (%)	38.6 ± 2.3	85.4 ± 2.8	46.7 ± 3.5	< 0.001*
Grip strength (Kgw)	23.5 ± 6.2	32.7 ± 9.8	8.3 ± 3.2	< 0.001*

Data are presented as *N* values or means ± standard deviations

^*^A *P* value of < 0.05 was considered statistically significant

## Discussion

Hermansdorfer and Kleinmen reported a case series of open repairs of chronic peripheral tears of the TFCC with favorable postoperative wrist functional outcomes in 1991 [18]. Cooney et al. reported favorable surgical outcomes of open TFCC repairs for Palmer type IB, IC, and ID lesions, with a patient satisfaction rate of 97%, in 1994 [19]. Zachee et al. became the first team to describe an arthroscopic-assisted TFCC repair method in 1993 [20], and Thomas et al. reported favorable surgical outcomes of arthroscopic repairs of Palmer type IB, IC, and ID peripheral TFCC tears, with a patient satisfaction rate of 89%, in 1997 [21]. In the present study, the outside-in method and a 1.3-mm suture anchor were used to treat patients with early foveal TFCC tears, and a satisfaction rate of 92.5% was achieved. In total, 189 of the 201 patients obtained “excellent” or “good” modified Mayo wrist scores. Therefore, the success rate of arthroscopic repair in the present study is comparable to that of open repair in the aforementioned previous reports. Arthroscopic repair also has the benefits of minimal invasiveness and accurate localization of foveal tears.

Physical examination allows for the accurate diagnosis of TFCC foveal tears. Palpation of the ballotable area of the ulnar bone frequently causes pain, and a positive TFCC stress test indicates a tear [22]. A positive TFCC stress test is associated with ulnar-sided wrist pain and occasional clicking sounds, reflecting the instability of a patient’s DRUJ. Foveal compression tests are also helpful indicators of foveal tears. Physical examination by careful and experienced medical practitioners can be used to easily diagnose TFCC problems.

MRI is up to 95% accurate in detecting TFCC tears [23]. In this study, all the patients underwent an MRI examination, which indicated TFCC foveal tears. MRI is sensitive for diagnosing lesions in our study and the literature reports [24–29], but arthroscopy appears to be the most accurate means of diagnosing the precise TFCC tear types.

PRP, which may provide an autologous source of platelets and contain alpha and dense granules rich in growth factors, has been used to accelerate tissue healing and repair in many areas of bone and soft tissue surgery for decades [30, 31]. The injection of PRP has also been commonly applied in the treatment of acute and chronic tendinopathies because it may promote cell viability and collagen synthesis by providing the growth factors TGF-β1 and PDGF-AB [32, 33]. In previous research, PRP improved the functional outcomes of treatment of wrist fractures or osteoarthritis [11]. To our knowledge, the present study is the first to apply PRP injection as an adjuvant therapy for TFCC repair. PRP injections may accelerate the healing of the TFCC and of subtle concomitant pathologies of the wrist joint, exerting a positive effect similar to its effect on lateral epicondylitis and rotator cuff injuries. Consequently, patients may achieve early and satisfactory recovery of their functions [34]. In future studies, researchers can compare the outcomes of patients who receive different doses of PRP.

This study has several limitations. First, we did not record or follow up some of the more subtle complications experienced by the patients, such as sensory nerve damage, wrist tightness, or mild discomfort. Second, patients with various types of TFCC lesions were assessed in this study, resulting in a heterogeneous sample. Furthermore, no comparative groups of patients who underwent treatment using other types of repair methods were included for comparison.

Most studies of foveal repair have involved the creation of a bony tunnel between the ulnar head and the foveal region. These methods require fluoroscopy to monitor the position of the tunnel and a special kit for targeting the fovea; furthermore, these methods do not entail debridement of both sides of a tear (the fovea and TFCC). However, in our method, debridement of both the foveal region and the deep layer of the TFCC can be achieved. The suture anchor also provides stability and strong suturing of the foveal region. In our study, most (92.5%) of the patients achieved “good” or “excellent” modified Mayo wrist scores, indicating that the postoperative functional outcomes of the patients who underwent TFCC repair through our method may be comparable to those of patients who underwent TFCC repair using other surgical methods in previous studies.

## Conclusions

According to the middle term surgical results of our study, we determined that arthroscopic repair of TFCC foveal tears with suture anchors after prompt diagnosis can effectively improve patients’ wrist function.

## Data Availability

All data generated or analyzed during this study are included in this published article.

## Abbreviations

TFCC: Traumatic triangular fibrocartilage complex
ECU: Extensor carpi ulnaris
DURJ: Distal radioulnar joint
PRP: Platelet-rich plasma
MRI: Magnetic resonance imaging
Atzei–EWAS: Atzei–European Wrist Arthroscopy Society
DASH: Disabilities of the Arm, Shoulder and Hand
